# Novel Findings in Breath-Holding Spells: A Cross-Sectional Study

**DOI:** 10.1097/MD.0000000000001150

**Published:** 2015-07-17

**Authors:** Seham F.A. Azab, Ahmed G. Siam, Safaa H. Saleh, Mona M. Elshafei, Wafaa F. Elsaeed, Mohamed A. Arafa, Eman A. Bendary, Elsayed M. Farag, Maha A.A. Basset, Sanaa M. Ismail, Osama M.A. Elazouni

**Affiliations:** From the Faculty of Medicine, Zagazig University, Zagazig City, Egypt (SFAA, AGS, SHS, MME, WFE, MAA, EAB, EMF, MAAB, SMI, OMAE).

## Abstract

The mechanism of breath-holding spells (BHS) is not fully understood and most probably multifactorial; so, this study was designed to clarify the pathophysiology of BHS through assessing some laboratory parameters and electrocardiographic (ECG) changes which might be contributing to the occurrence of the attacks. Another aim of the study was to evaluate the differences in the pathophysiology between pallid and cyanotic types of BHS.

This was a prospective study performed in Zagazig University Hospitals. Seventy-six children diagnosed with BHS were included as follows: 32 children with cyanotic BHS, 14 children with pallid BHS, and 30 healthy children as a control group. All children were subjected to the following: full history taking, clinical examination, and laboratory work up in the form of CBC, serum iron, ferritin, and zinc levels. Twenty-four hours ambulatory ECG (Holter) recording was also performed.

No significant statistical difference was found between cyanotic and pallid groups regarding family history of BHS, severity, and precipitating factors of the attacks. Frequent runs of respiratory sinus arrhythmia (RSA) during 24 hours ECG were significantly higher in children with BHS; the frequency of RSA was significantly correlated with the frequency (severity) of the attacks. Low serum ferritin was significantly associated with BHS groups but not correlated with the severity of the attacks.

Autonomic dysregulation evidenced by frequent RSA is considered to be an important cause of BHS in children and is correlated with the frequency of the attacks. Low serum ferritin is additional factor in the pathophysiology. Both pallid and cyanotic BHS are suggested to be types of the same disease sharing the same pathophysiology.

## INTRODUCTION

Breath holding spells (BHS) is a common problem in children, and particularly so in infants and is a frightening experience for the parents.^1,2^ BHS is apparently due to acute cerebral hypoxia, and the child recovers spontaneously after a period of unconsciousness and sometimes opisthotonic posturing.^3^ BHS may occur in children with normal neurological development, and usually have no impact on the subsequent development.^4^ The diagnosis is based on stereotyped sequence of clinical events that are triggered by mild trauma/emotional upset/frustration, which is followed by crying leading to noiseless state of expiration associated with color changes either cyanosis or pallor and ultimately loss of consciousness.^4,5^ The spells begin most commonly during the first 12 months of life and almost always by 2 years of age. Most children outgrow those spells approximately at the age of 6 years.^6^ Rarely BHS continue and are replaced by vasovagal attacks.^7^

Two types of BHS are present based on the color of the child during the apneic episode following the end of prolonged expiration either pale (pallid attacks) or blue (cyanotic attacks); rarely both types may occur in the same child (mixed type).^1,4,8^

The mechanism of BHS is not fully understood and most probably multifactorial.^7,9,10^ Iron deficiency anemia is claimed to be associated with the occurrence of BHS supported by the response of some cases to iron therapy; associated zinc deficiency may be an additional factor.^10,11^ Autonomic dysregulation that leads to alteration in cardiac function and simultaneous decrease in cerebral blood flow is an important risk factor,^12,13^ so electrocardiogram (ECG) should be strongly considered in any patient with BHS.^14^ In this study, we aimed to clarify the pathophysiology of breath-holding spells by assessing factors claimed to be associated with the attacks including laboratory parameters and continuous ECG monitoring and to identify differences in the pathophysiology between pallid and cyanotic BHS.

## METHODS

This was a prospective cross-sectional study performed in Zagazig University Hospitals from December 2012 to January 2014. A total of 76 children diagnosed with BHS at the child neurology out-patient clinics were included and assessed at the Pediatrics, Neurology and Cardiology Departments of the same Hospitals. Their age ranged from 15 to 48 months (mean 31 months). We divided our cases into 3 groups:

1. Group I: 32 patients with cyanotic BHS.
2. Group II: 14 patients with pallid BHS.
3. Group III: 30 healthy children, of comparable age and sex; who attended Pediatric Department for preoperative evaluation for elective surgery, were enrolled as a control group.

### Exclusion Criteria

Children with primary cardiac or central nervous system disease were not included as well as any child with uncertain history of the type of BHS or suspected mixed type. Any child with possibility of seizure was subjected to EEG assessment and children with abnormal EEG were also excluded. Initial basic ECG rhythm strip for 30 seconds was done to exclude prolonged QT syndrome.

All children were subjected to the following:

- Full history taking including age, sex, parental consanguinity, and thorough clinical examination.
- Detailed history of the attacks with stress on family history, age of onset, triggering factors, and frequency of the attacks. Attacks were categorized according to average frequency of occurrence:
- Mild frequency → <one attack/week
- Moderate frequency → 1–3 attacks/week
- High frequency → >3 attacks/week
- Laboratory investigations including RBC's count and hemoglobin level (to assess anemia), serum iron (by colorimeteric chromazuol B),^15^ serum ferritin (by electrochemluminescence using elecsys and cobas (2010) immunoassay analyzers),^16^ and serum zinc (by atomic absorption spectrophotometer [GBC GF 3000).^17^

Laboratory results were described in the form of normal, low or high according to their reference range for each age.

Electrocardiographic (ECG) study: 24 hours ambulatory ECG recording was done for all children using (VX3 series E) recorders analyzed by H7000 Holter software for detection of changes in ECG during and in-between the attacks of BHS.

### Ethics

Permission was obtained from the ethical committee of Zagazig University and the head of Pediatrics, Neurology and Cardiology Departments before the beginning of the study. An informed written consent was obtained from the care givers of all children included in the study. The study was done according to the rules of the Local Ethics Committee of Faculty of Medicine, Zagazig University, Egypt.

### Statistical Methods

SPSS version 19 and EPI-info Version 6.04 were used for data analysis. The data are expressed as the mean ± SD or median (min–max) where appropriate. Test selection was based on evaluating the variables for normal distribution using the Shapiro–Wilk test. If the variables had a normal distribution, Student *t* test was used. If the variable did not have a normal distribution, the analysis was done using the Mann–Whitney *U* test. Categorical data were evaluated by Pearson *χ*^2^ test. Statistical correlations were calculated by Pearson correlation test. *P* < 0.05 was considered significant.

## RESULTS

Our study included 46 patients with BHS (32 patients with cyanotic BHS and 14 patients with pallid BHS, their age ranged from 15 to 48 months (mean 31 months), 27 males and 19 females) and 30 healthy control subjects whose characteristics are listed in Table 1. The control group were age and sex matched to children with BHS with no statistical difference regarding consanguineous parental marriage.

**TABLE 1:**
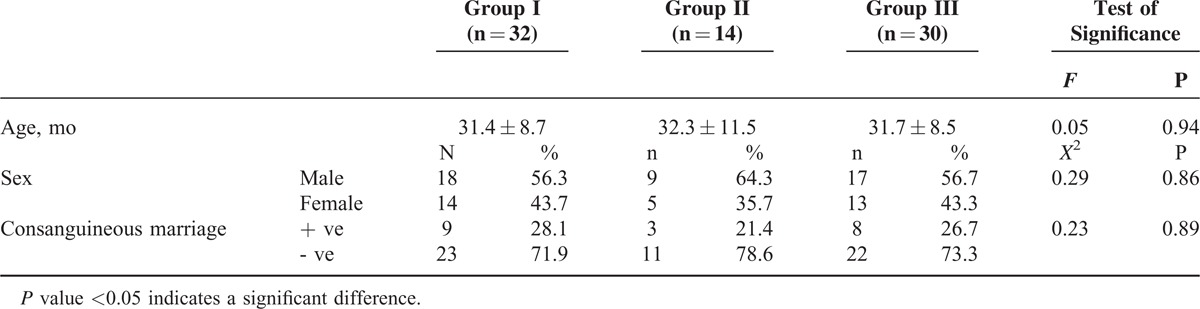
Characteristics of the Studied Population

Of note, there was no significant difference between patients with cyanotic spells (group I) and those with pallid spells (group II) regarding the age of onset of the attacks, the presence of similar attacks in the family, the frequency of the attacks, and the nature of the precipitating factors (all *P* > 0.05; Table 2).

**TABLE 2:**
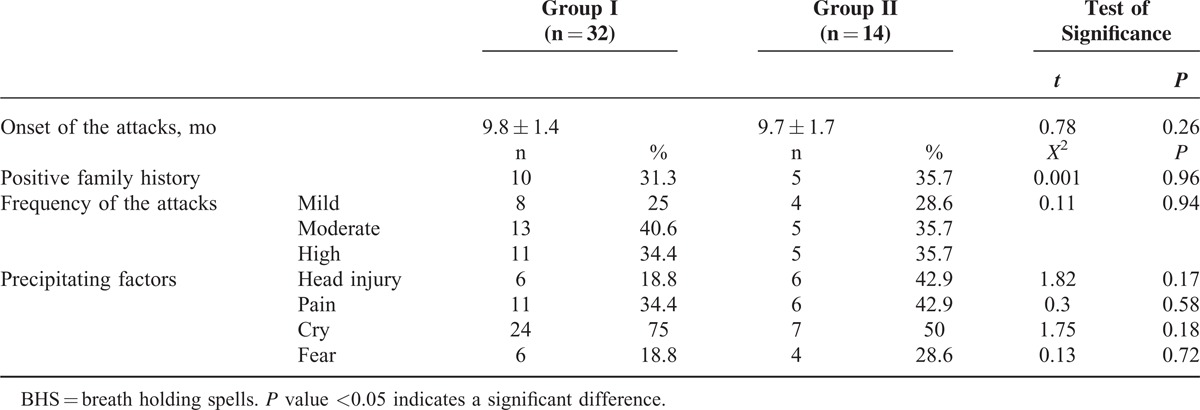
Features of BHS in Cyanotic and Pallid Types

Table 3 shows the differences between patients with cyanotic spells (group I) and those with pallid spells (group II) and normal control (group III) as regards some laboratory parameters claimed to be affected in patients with BHS (the laboratory findings were set as normal, high, or low according to the normal parameters for each case according to their age and sex at the time of sampling); Serum ferritin was significantly lower in patients with BHS compared with the control group (*P* < 0.05). However, presence of anemia, lower serum iron, or zinc were not found to be a differentiated test between all groups (all *P* > 0.05; Table 3).

**TABLE 3:**
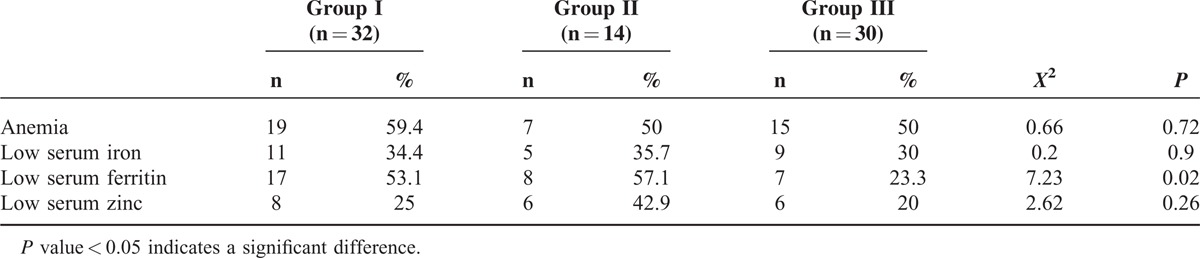
Laboratory Findings of the Studied Population

The frequency of respiratory sinus arrhythmia (RSA) in 24 hours ECG monitoring among different groups was classified into few (<5/24 hrs), moderate (5–10/24 hrs), and frequent (>10/24 hrs). Our data showed that cases with BHS had significant higher runs of respiratory sinus arrhythmia in comparison with the control group (*P* = 0.001; Table 4).

**TABLE 4:**
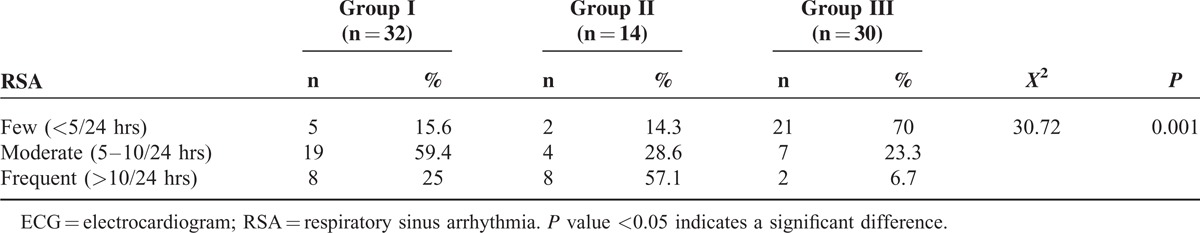
Frequency of RSA in 24 hours ECG Monitoring Among Studied Groups

**FIGURE 1:**
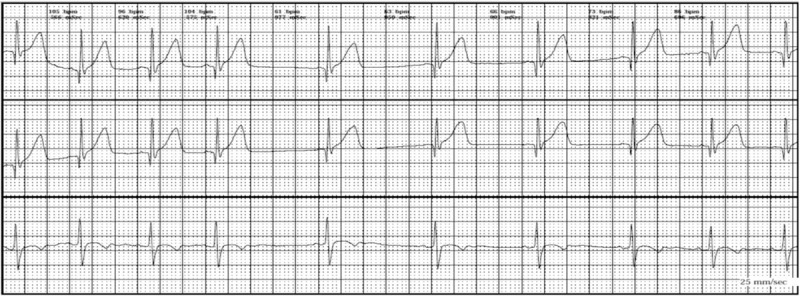
Respiratory sinus arrhythmia.

**FIGURE 2:**
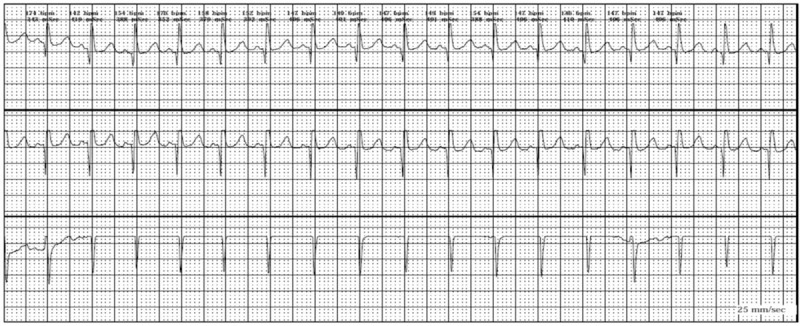
Sinus tachycardia.

**FIGURE 3:**
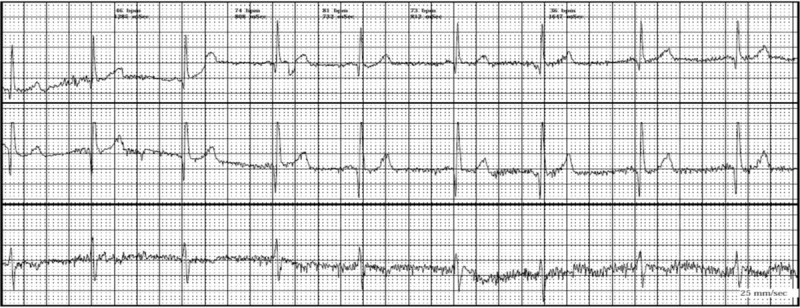
Sinus bradycardia.

Of note, 10 (21.7%) of BHS patients experienced breath-holding attacks during 24 hours ECG recording (7 patients experienced cyanotic BHS and 3 patients experienced pallid BHS). Moreover, the most common findings to appear during the attack were RSA (40%) (Figure 1) and sinus tachycardia (40%) (Figure 2) followed by sinus bradycardia (10%) (Figure 3) and asystole (10%) (Table 5)

**TABLE 5:**
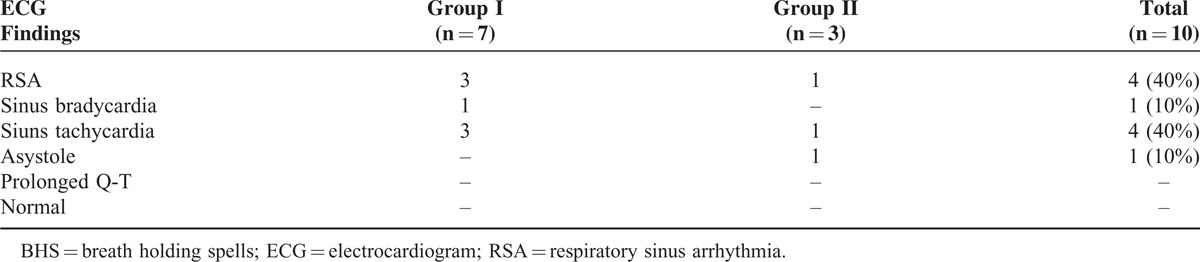
ECG Changes During the BHS Attacks

Our data showed a significant relation between frequency of the BHS attacks and frequency of RSA in 24 hours ECG recording (*P* = 0.007; Table 6a). Meanwhile, there was no significant relation between the frequency of the BHS attacks and low serum ferritin (P = 0.29; Table 6b). Our study revealed a significant correlation between the frequency of RSA in Holter monitoring and the frequency of BHS obtained from parental history (*P* < 0.001). On the other hand, low serum ferritin despite being significantly associated with BHS but was not correlated with the frequency of the attacks (*P* > 0.05; Table 6c).

**TABLE 6:**
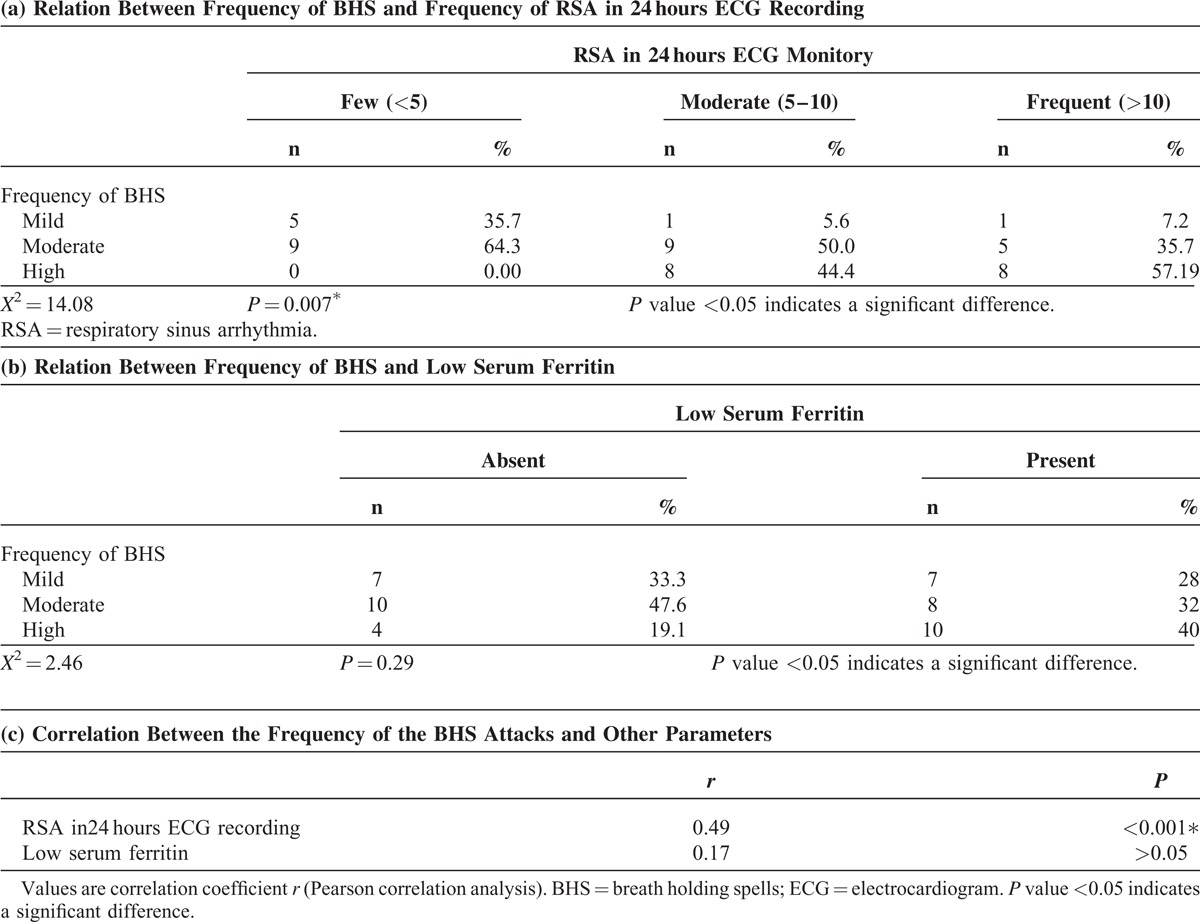
Factors Affecting Frequency of BHS

## DISCUSSION

The pathophysiologic mechanism of BHS remains controversial and no study had identified the exact etiology of the attacks.^6,9^ In our study, the average age of onset of BHS was 9.8 and 9.7 months for cyanotic and pallid groups, respectively, which is not far from other studies which stated that most cases of BHS are manifested before the first birthday.^5,18,19^ Similar attacks of BHS in first degree relative (positive family history) were present in about one-third of cases of BHS (31.3% for cyanotic and 35.7% for pallid BHS), which is consistent with multiple studies.^4–6^ DiMario et al explained this considerable positive family history by the autosomal-dominant inheritance with incomplete penetrance of BHS.^20^ However, as no specific gene had been identified for inheritance of BHS, the presence of family history may be related to the inheritance of the cause of BHS like autonomic dysfunction rather than BHS themselves.^13,20,21^

Multiple precipitating factors were associated with the attacks, of which, cry was the most common event preceding cyanotic (75%) and pallid (50%) BHS. However, no single factor showed statistical significant association with either type of BHS.

Children with BHS showed no significant statistical difference regarding anemia and low serum iron when compared with the control group. Multiple studies had reported a high incidence of iron deficiency anemia in children suffering from BHS.^6,9,11,12,22^ No other types of anemia were claimed to be associated with BHS in any study, so, it is obvious that the problem is related to “iron” and not to “anemia”; as body iron is better assessed by serum ferritin rather than serum iron which is affected by many factors.^23^

Low serum zinc was not associated with increased risk of BHS. A single study performed by Gencgonul et al in Turkey (2002) had suggested a role of zinc deficiency in the pathogenesis of BHS^11^; however, the Gencgonul study only suggested a role of zinc deficiency in association with iron deficiency—and not zinc alone—in the pathogenesis of BHS; as zinc deficiency is a common association in iron deficiency anemia our results regarding this issue seem logic.

24 hours ECG monitoring was done for all children subjected to our study. Respiratory sinus arrhythmia (RSA) was the only significant finding in 24 hours ECG monitoring in cases with BHS; most cases of either cyanotic or pallid BHS showed higher runs of respiratory sinus arrhythmia compared with the control group. Respiratory sinus arrhythmia was identified if PP intervals were present that exceeded the previous PP by more than 10%.^24^

A study of DiMario et al had suggested the same association with pallid and not with cyanotic BHS,^21^ the difference between the results of DiMario et al and ours may be attributed to the fewer number of cases in their study (41 vs. 76); also, the 24 hours ECG recording performed to our studied children is probably more accurate than DiMario et al study who performed a 5-min, ordinary ECG.

Only 10 cases experienced an attack of BHS during 24 hours ECG recording and revealed RSA and sinus tachycardia to be the most common findings, although statistical analysis could not be performed due to small sample size. Among the 10 patients who experienced an attack during Holter ECG monitoring, no data of prolonged QT interval were obtained. Cases with persistent prolonged Q-T were not included in our study according to our exclusion criteria as prolonged Q-T syndrome associated syncope is a serious condition which may need interference up to pacemaker implantation.^4,7,10,19,25^ On the other hand, Akalin et al (2004) had linked the BHS with increased QT dispersion in ECG^12^ but their study did not exclude patients of prolonged QT syndrome which may extend their study outside the scope of BHS.

In our study, we categorized our patients according to their frequency of attacks.

Sartori et al^26^ had considered serious clinical events as a measure for severity of BHS. These phenomena included significant bradycardia or prolonged asystole; prolonged loss of consciousness; severe generalized stiffness, opisthotonus, clonic, myoclonic or tonic–clonic jerks; epileptic seizures or even status epilepticus. The parameters used in the literature to address case severity were the same regarding either pallid or cyanotic types. Categorization according to serious clinical phenomena seems more logic when a decision of cardiac pacing is required as the use of frequency of attacks without other severity parameters is not sufficient. This point was a limitation of our study regarding the decision of cardiac pacing.

Sartori et al found that most of severe cases that required cardiac pacing were related to the pallid type. They reported that 48 cases with BHS required cardiac implantation. Twenty-seven cases were of the pallid type, 18 cases had mixed forms, and only 2 cases were cyanotic. These cumulative data represent that pallid cases were more severe.

Our study revealed a significant correlation between the frequency of RSA in Holter monitoring and the frequency of BHS obtained from parental history, which is an important evidence indicating the basic role of cardiac rhythm abnormality in the pathophysiology of BHS and strengthen the results of other studies which concluded that autonomic dysregulation is the primary abnormality in children with BHS that leads to defective cerebral blood flow followed by the sequence of events observed.^12,13,21^ On the other side, low serum ferritin despite being significantly associated with BHS but was not correlated with the frequency of the attacks which may indicate that iron deficiency assessed by low serum ferritin is not the primary pathophysiology of BHS but an additional factor contributed to the etiology. The association of iron deficiency and BHS may be related to the following:

1. Iron deficiency is usually associated with irritability and excess crying^5,6^ which is the commonest triggering factor for the attacks.
2. Iron deficiency may lead to catecolamine disruption and subsequent autonomic dysregulation^6,27^ and this was proved by Orri et al who reported an improvement of autonomic dysregulation after iron supply in 3 children with BHS.^27^

Last, among all clinical, laboratory, and ECG data obtained from the present study no significant difference was found between cyanotic and pallid BHS which indicates that both types of BHS share the same pathophysiologic mechanism, that is, pallid and cyanotic BHS are “1” and not “2” diseases. This conclusion is not in agreement with some studies which postulated that the pathophysiology of pallid and cyanotic spells is not the same.^7,14,21^ Again, the use of 24 hours ECG recording in our study which is more accurate than ordinary ECG may add more confidence to our results. Moreover, the presence of a considerable percentage of children who experienced both types of BHS, that is, mixed type, may indicate that the pathogenesis of both types may be similar.

Differences between cyanotic and pallid BHS regarding sequence of events and color changes might be explained by the dominant autonomic dysregulatory component which may be sympathetic over activity in cyanotic and parasympathetic in pallid BHS.^4^ This refers to a difference in the type of patient (regarding variable autonomic predominance) rather than distinct disease entities.

To the best of our knowledge, this is the first study that performed a Holter's monitoring for children with BHS and; all published reviews of ECG interpretation in BHS depend on ordinary short ECG strips. However, the small sample size was one of our limitations in this study; we suggest that multicenter approaches may be necessary to attain larger sample sizes. Finally, future longitudinal cohort studies are recommended to validate the current findings taking into consideration the different degrees of BHA severity which was another limitation in our study.

## Conclusion

Autonomic dysregulation evidenced by frequent RSA is considered to be an important cause of BHS in children and is correlated with the frequency of the attacks. Low serum ferritin is additional factor in the pathophysiology. Both pallid and cyanotic BHS are suggested to be types of the same disease sharing the same pathophysiology.
